# Identifying risk factors associated with smear positivity of pulmonary tuberculosis in Kazakhstan

**DOI:** 10.1371/journal.pone.0172942

**Published:** 2017-03-01

**Authors:** Sabrina Hermosilla, Paul You, Angela Aifah, Tleukhan Abildayev, Ainur Akilzhanova, Ulan Kozhamkulov, Talgat Muminov, Meruert Darisheva, Baurzhan Zhussupov, Assel Terlikbayeva, Nabila El-Bassel, Neil Schluger

**Affiliations:** 1 Columbia University Mailman School of Public Health, Department of Epidemiology, New York, NY, United States of America; 2 Columbia University School of Social Work, 1255 Amsterdam Avenue, New York, NY, United States of America; 3 National Center for Tuberculosis Problems, Almaty, Kazakhstan; 4 Center for Life Sciences, Nazarbayev University, 53 Kabanbay Batyr Ave., Astana, Republic of Kazakhstan; 5 National Association for TB Specialists, Almaty, Kazakhstan; 6 Columbia University Global Health Research Center of Central Asia, Almaty, Kazakhstan; University of South Africa, UNITED STATES

## Abstract

**Background:**

Sputum smear-positive tuberculosis (TB) patients have a high risk of transmission and are of great epidemiological and infection control significance. Little is known about the smear-positive populations in high TB burden regions, such as Kazakhstan. The objective of this study is to characterize the smear-positive population in Kazakhstan and identify associated modifiable risk factors.

**Methods:**

Data on incident TB cases’ (identified between April 2012 and March 2014) socio-demographic, risk behavior, and comorbidity characteristics were collected in four regions of Kazakhstan through structured survey and medical record review. We used multivariable logistic regression to determine factors associated with smear positivity.

**Results:**

Of the total sample, 193 (34.3%) of the 562 study participants tested smear-positive. In the final adjusted multivariable logistic regression model, sex (adjusted odds ratio (aOR) = 2.0, 95% CI:1.3–3.1, p < 0.01), incarceration (aOR = 3.6, 95% CI:1.2–11.1, p = 0.03), alcohol dependence (aOR = 2.6, 95% CI:1.2–5.7, p = 0.02), diabetes (aOR = 5.0, 95% CI:2.4–10.7, p < 0.01), and physician access (aOR = 2.7, 95% CI:1.3–5.5p < 0.01) were associated with smear-positivity.

**Conclusions:**

Incarceration, alcohol dependence, diabetes, and physician access are associated with smear positivity among incident TB cases in Kazakhstan. To stem the TB epidemic, screening, treatment and prevention policies should address these factors.

## Introduction

Tuberculosis (TB) remains one of the world’s most burdensome communicable diseases[1]. Despite the gradual decline in TB morbidity and mortality over recent years, the control and elimination of the disease remains a significant challenge[1]. Kazakhstan, an upper middle-income country, is currently characterized by the World Health Organization (WHO) as a high TB priority country and has the second highest multidrug-resistant TB (MDR-TB) burden globally[2].

*Mycobacterium tuberculosis* infection is associated with modifiable factors including incarceration[3], smoking[4], alcohol dependence[5], drug use history[6], low body mass index (BMI; as both a potential risk factor and symptom of infection)[7], diabetes [7], Hepatitis C virus (HCV)[8, 9], HIV status [10], and depression[11]. While descriptive epidemiological studies have documented the TB burden in Kazakhstan[12], few have linked these factors to increased infectiousness or identified highly infectious sub-populations.

For low- and middle-income countries, including Kazakhstan, sputum smear microscopy using unconcentrated samples with Ziehl-Neelsen staining is the cornerstone of TB diagnosis[13]. This test is rapid, simple, inexpensive, and widely available in TB-endemic countries. Detection of tubercle bacilli in sputum samples through microscopy indicates that the patient is capable of TB transmission[14]. Although those with smear-negative, culture-positive pulmonary TB are capable of infecting close contacts, smear-positive patients are considered most infectious[14]. Smear positivity among incident pulmonary TB cases is associated, in low MDR-TB burden contexts, with risk factors such as: alcohol abuse[15], homelessness[15], incarceration[15], unemployment[15], male gender[15], and urban residence[15]; and health-related outcomes such as HIV status (both high[16] and low positive prevalence[17]), high mortality[18], and poor TB treatment outcomes[15].

To our knowledge there are no studies exploring associations between modifiable risk factors and smear-positivity in a high MDR-TB setting. Given the higher risk of transmission, smear-positive patients are of great epidemiological importance[19]. We conducted a cross-sectional study to examine factors associated with sputum smear positivity, among pulmonary TB patients, in Kazakhstan. We hypothesize that socio-demographic, behavioral, and co-morbidity risk factors play an important role in increasing an individual’s odds of sputum smear positivity.

## Materials and methods

### Data collection and study population

All pulmonary TB incident cases diagnosed between April 2012 and March 2014 registered in the National Tuberculosis Program (NTP) in Almaty (Almaty city and state), Kostanay, and Kyzylorda, Kazakhstan—representing a cross-section of high, medium, and low burden regions[12]—were invited to participate in our study through a combination of TB clinician pre-screening, research staff screening, and electronic medical record review. Participant data were collected through an in-person 60-minute audio computer assisted questionnaire conducted from June 2012 to May 2014 and a targeted review of their clinical medical records. The study instrument was designed in English, from scales previously used in the region, translated and pilot tested in Russian and Kazakh, then back translated to English. Once finalized, the questionnaire was programmed and presented in Russian or Kazakh through DatStat, a software providing video presentation of questions and responses. All questionnaires were administered in private rooms with research assistants available, if necessary.

During the study period, 744 incident TB cases were registered by the NTP. Other study eligibility criteria includes: (1) age– 18 years or older; (2) at permanent residence address for three or more months; (3) sampling requirements for parent longitudinal study (not relevant to current analysis), including availability of a pulmonary TB negative household adult control; (4) Russian or Kazakh language fluency; and (5) absence of apparent severe mental disorders that may impede their ability to consent and complete the questionnaire. Upon completing the primary screening, 566 (76.0% of approached) participants met the eligibility criteria and 562 participants agreed to participate.

### Measures

#### Outcome definition: Incident pulmonary tuberculosis positive definition and sputum smear microscopy results

All participants were incident pulmonary TB cases. In this study, a positive pulmonary TB diagnosis was defined as any suspected TB case that had at least one positive sputum smear microscopy result, or a negative sputum smear microscopy result, but presented clinical or radiographic symptoms consistent with TB that responded to drug treatment, based on current Kazakh national treatment guidelines[20]. The main outcome of interest for this analysis, sputum smear positivity, was assessed through microscopy results that were collected retrospectively from participant’s medical records and documented on individual registration cards that were completed for each participant. Individuals with at least one positive smear result were considered sputum smear-positive.

#### Socio-demographics

Socio-demographic characteristics were collected, including gender, age (years old), nationality (Kazakh or other), country of birth (Kazakhstan or other), marital status, highest education received, employment status, monthly income, living in an urban or rural setting, and social support. Social support was measured as a continuous variable by employing the Multidimensional Scale of Perceived Social Support (MSPSS), previously used in the region[21].

#### TB behavioral risk factors and comorbidity

Behavioral factors previously shown to be associated with TB were collected, including criminal history (ever having been incarcerated), smoking status (smoked tobacco in the past 12 months), alcohol dependence, and history of drug use (positive endorsement to item “Have you ever used drugs?”). Alcohol dependence was measured by the validated CAGE questionnaire, which consists of four questions[22]. Alcohol dependence was defined as answering “yes” to two or more of these questions, or answering “yes” to the question “Have you ever felt you needed a drink first thing in the morning to steady your nerves or to get rid of a hangover (eye-opener)[22]?” Access to health services (provided to all Kazakh nationals for free) was approximated by the participant endorsement of the item “Do you have a regular doctor to see when you are sick?” While all participants were sampled from a healthcare-seeking population, which by definition seeks services more than those outside care, even within the care-seeking population differential service utilization could influence diagnostic patterns and thus smear positivity. Participants were asked to self-report comorbid factors including diabetes, anemia, hepatitis C virus (HCV), HIV status, and depression. Depression was scored as a continuous variable using the Brief Symptom Inventory–Depression subscale[23]. BMI at diagnosis was collected from participants’ clinical medical records.

### Statistical analysis

First, we conducted descriptive univariate analyses to illustrate relevant characteristics of the TB-positive population, informed by a thorough literature review exploring factors driving sputum smear positivity. Second, we conducted bivariate analyses to identify statistically significant differences in socio-demographic, behavioral, and comorbidity factors between smear-positive and smear-negative populations by performing Chi-Square and Fisher’s exact tests for categorical variables and Student t-tests (Wilcoxon for variables non-normally distributed, as identified through Shapio-Wilk normality tests) for continuous variables. Third, simple bivariate logistic regressions were performed to identify potentially significant (p < 0.10) associations between socio-demographic, behavioral, and comorbidity factors and smear positivity. Fourth, the final multivariable logistic regression analysis estimated the relationship between sputum smear status and statistically significant factors (p < 0.10) from the previous simple bivariate regression analysis and factors deemed epidemiologically relevant based on the extant literature (e.g. tobacco usage, social support, health system access, and HCV). Once estimated, we performed diagnostics of the final multivariable logistic regression model to explore: model fit (Hosmer-Lemeshow goodness of fit), the potential for collinearity among included variables, and residual distribution. Significance for the final model was defined as p value < 0.05. All analyses were conducted in SAS 9.4.

### Ethical consideration

All identifying information were removed before performing analysis. Ethical review was approved by Institutional Review Boards from Columbia University (Protocol AAAJ8510), Center for Life Sciences of Nazarbayev University (Protocol 2, 24 at April 17, 2012 meeting) and the National TB Center of Kazakhstan (March 19, 2012 meeting). Written informed consent was acquired from all participants, stored in a secure study location, and was approved by Institutional Review Boards.

## Results

### Participant characteristics

744 TB-positive index cases were identified from 2012 to 2014, of which 562 (75.5%) agreed to complete the questionnaire. Participant’s mean age was 35 (SD = 13.4) years, ranging from 18 to 83 years. Table 1 shows the study population demographic characteristics, risk factors, and comorbidities of all TB-positive index cases and stratified by smear-positive diagnostic status. The study population was 55.0% male, with 61.7% currently married and 76.9% Kazakh nationals. The majority (80.6%) of the population were employed full-time and 92.7% had attended high school or more of formal schooling, consistent with UNICEF’s description of the overall Kazakh population in 2012[24].

**Table 1:** Study population characteristics among 562 total and smear-positive participants, Kazakhstan, 2012–2014.

	Smear+ (193)		Smear- (355)		Total (562)		
	% (n)	95% CI	% (n)	95% CI	% (n)	95% CI	p-value
**Socio-demographic**							
Sex, male	63.73% (123)**	56.92%–70.53%	50.7% (180)	45.48%–55.92%	54.98% (309)	50.86%–59.11%	0.003
Age, mean (SD)	36.5 (13.4)	34.5–38.3	34.7 (13.0)	33.3–36.0	35 (13.1)	34.2–36.4	0.118
Employed	80.83% (156)	75.258%–86.4%	80.56% (286)	76.43%–84.69%	80.60% (453)	77.33%–83.88%	0.94
Highest education							
Primary school	0.52% (1)	0.00%–1.53%	0.28% (1)	0.00%–0.83%	0.36% (2)	0.00%–0.84%	0.6095
Secondary school	8.81% (17)	4.79%–12.81%	6.20% (22)	3.68%–8.71%	6.94% (39)	4.83%–9.04%	
High school	28.50% (55)	22.10%–34.88%	31.83% (113)	26.97%–36.69%	30.60% (172)	26.78%–34.42%	
Vocational education	44.04% (85)	37.01%–51.06%	42.54% (151)	37.37%–47.69%	43.06% (242)	38.95%–47.16%	
Incomplete higher education	5.18% (10)	2.04%–8.31%	3.38% (12)	1.49%–5.26%	4.27% (24)	2.59%–5.94%	
Higher education	12.95% (25)	8.20%–17.70%	15.77% (56)	11.97%–19.57%	14.77% (83)	11.82%–17.71%	
Nationality							
Kazakh	70.98% (137)*	64.56%–77.40%	80.00% (284)	75.82%–84.17%	76.87% (432)	73.37%–80.37%	0.017
Other	29.02% (56)	22.59%–35.43%	20.00% (71)	15.82%–24.17%	23.13% (130)	19.63%–26.62%	
Marital Status, Married	58.55% (113)	51.57%–65.52%	62.82% (223)	57.77%–67.86%	61.74% (347)	57.71%–65.77%	0.327
Monthly income, mean(SD)	45474 (53896)	37823–53127	38727 (58902)	32579–44876	40781 (56970)	36061–45501	0.218
Place of birth							
Kazakh	89.64% (173)	85.32%–93.95%	91.55% (325)	88.64%–94.45%	91.10% (512)	88.74%–93.46%	0.458
Other	10.36% (20)	6.04%–14.67%	8.45 (30)	5.54%–11.35%	8.90% (50)	6.53%–11.25%	
Urban	52.85% (102)*	45.78%–59.91%	41.48% (146)	36.31%–46.64%	45.77% (254)	41.61%–49.92%	0.011
Study Site							
Almaty city	18.65% (36)	13.14%–24.16%	4.79% (17)	2.56%–7.01%	9.96% (56)	7.48%–12.44%	<0.001
Almaty area	23.32% (45)	17.33%–29.30%	37.75% (134)	32.68%–42.80%	32.92% (185)	29.02%–36.81%	
Kyzylorda	29.02% (56)	22.59%–35.43%	33.52% (119)	28.59%–38.44%	31.14% (175)	27.29%–34.97%	
Kostanay	29.02% (56)	22.59%–35.43%	23.94% (85)	19.49%–28.39%	25.98% (146)	22.34%–29.61%	
**Behavioral**							
Ever incarcerated	6.81% (13)**	3.22%–10.38%	1.98% (7)	0.52%–3.43%	3.76% (21)	2.18%–5.34%	0.004
Smoked tobacco in the past 12 months	22.41% (39)	16.19%–28.63%	15.79% (51)	11.79%–19.77%	18.24% (93)	14.87%–21.60%	0.067
Ever consumed alcohol	55.96% (108)*	48.93%–62.98%	47.04% (167)	41.83%–52.25%	49.64% (279)	45.50%–53.79%	0.046
Alcohol dependence^§^	17.10% (33)***	11.77%–22.42%	6.76% (24)	4.14%–9.38%	10.32% (58)	7.80%–12.84%	<0.001
Ever used drugs	9.33% (18)**	5.21%–13.44%	3.10% (11)	1.29%–4.90%	5.34% (30)	3.47%–7.20%	0.002
Social Support Score, mean (SD)	64.6 (10.6)	63.1–66.1	64.7 (10.2)	63.6–65.7	64.6 (2.88)	63.7–65.4	0.81
Have a doctor	90.16% (174)	85.93%–94.37%	85.07% (302)	81.35%–88.78%	86.30% (485)	83.44%–89.15%	0.092
**Comorbidity**							
BMI, mean (SD)	20.8 (3.8)	20.2–21.3	21.4 (2.6)	21.0–21.8	21.2 (3.7)	20.9–21.5	0.005
Ever diagnosed with diabetes	13.09% (25)***	8.29%–17.88%	4.23% (15)	2.12%–6.32%	7.14% (40)	5.00%–9.28%	<0.001
Ever diagnosed with anemia	11.46% (22)	6.93%–15.97%	15.54% (55)	11.75%–19.32%	14.11% (79)	11.21%–16.99%	0.191
Ever diagnosed with HCV	3.63% (7)*	0.98%–6.27%	1.13% (4)	0.02%–2.22%	2.14% (12)	0.93%–3.33%	0.046
HIV Status, Positive	2.60% (4)	0.07%–5.11%	0.97% (3)	0.00%–2.06%	1.48% (7)	0.38%–2.57%	0.175
Depression Score, mean (SD)	1.64 (2.84)	1.23–2.045	1.60 (3.03)	1.28–1.91	1.62 (2.99)	1.37–1.86	0.486

Notes. HCV: Hepatitis C virus; all *p* values refer to comparison between smear-positive and smear-negative subjects only: Chi-Square and Fisher’s exact tests for categorical variables and Student t-tests for continuous variables

* *p*<0.05.

** *p*<0.01

*** *p*<0.001

^§^ as measured by the CAGE.

### Behavioral risk factors and comorbidity

Participants reported: having a regular doctor they could see when sick (86.3%); low levels of alcohol consumption (49.6% ever consumed alcohol) and dependence (10.3% based on CAGE results); and moderate levels of tobacco smoking (18.2% in the past 12 months). Diabetes (7.1% ever diagnosed) and HCV (2.1% ever diagnosed) were the most commonly reported comorbid conditions.

### Smear-positive risk factors

A third, 193 (34.3%), of the study participants were smear-positive with 14 (2.5%) participants having invalid or no smear results. Through bivariate analyses the smear-positive population had significantly more males (63.7%, p = 0.003) and less Kazakh nationals (71.0%, p = 0.017) than those who were reported as smear-negative. Education level, employment and marital status were not statistically different between the two smear categories. Smear-positive patients consistently reported significantly higher rates of incarceration (6.8% vs. 3.8%), recently smoked more (22.4% vs. 18.2%), were more dependent on alcohol (17.1% vs. 10.3%), and more frequently lived in urban settings (52.9% vs. 45.8%). Most participants, irrespective of smear status, reported high levels (64.6 mean score) of social support from either family members or friends. Smear-positive cases reported increased rates of diabetes (13.1%) and HCV (3.6%). No significant difference in BMI was observed. Similarly, HIV (1.5%) and depression (1.62) did not differ significantly by smear status.

Table 2 shows the unadjusted (OR) and adjusted odds ratios (aOR) and their 95% confidence intervals generated by simple logistic regression and multivariable logistic regression analysis, respectively. Upon removing those with missing values, our final model included 480 participants. The final adjusted multivariable logistic regression model, identifying independent associations with sputum smear positivity, showed significant associations between smear positivity and male gender [aOR 1.97 (1.25, 3.11)], Almaty area [aOR 0.15 (0.07, 0.35)], Kyzylorda [aOR 0.22 (0.10, 0.51)], and Kostanay [aOR 0.29 (0.13, 0.64)] study sites, ever being incarcerated [aOR 3.62 (1.18, 11.13)], alcohol dependence [aOR 2.61 (1.20, 5.69)], having a doctor to see when ill [aOR 2.69 (1.32, 5.49)], and diabetes [aOR 5.02 (2.35, 10.71)]. Kazakh nationality [aOR 0.70 (0.40, 1.22)], setting [aOR 1.07 (0.68, 1.69)], recently smoked tobacco [aOR 0.57 (0.28, 1.13)], ever-used drugs [aOR 1.48 (0.58, 3.76)], and HCV [aOR 4.28 (0.85, 21.57)] were not statistically significant. The final model indicates a changed protective effect of living in Almaty area, Kyzylorda, and Kostanay when compared to Almaty city.

**Table 2:** Crude and Adjusted Associations with Smear-positive Among 480 Pulmonary Tuberculosis Cases, Kazakhstan, 2012–2014.

	Unadjusted		Adjusted	
	OR (95% CI)	*p*-value	aOR (95% CI)	*p*-value
**Socio-demographic**				
Sex, male (ref. female)	1.71 (1.19, 2.45)	0.004**	1.97 (1.25, 3.11)	0.004**
Nationality, Kazakh (ref. other)	0.61 (0.41, 0.92)	0.017*	0.70 (0.40, 1.22)	0.21
Study Site				
	Almaty city	ref		ref	
Almaty area	6.32 (3.23, 12.30)	<0.001***	0.15 (0.07, 0.35)	<0.001***
Kyzylorda	4.50 (2.33, 8.69)	<0.001***	0.22 (0.10, 0.51)	<0.001***
Kostanay	3.21 (1.64, 6.27)	<0.001***	0.29 (0.13, 0.64)	0.002**
Urban (ref. rural)	1.58 (1.11, 2.25)	0.011*	1.07 (0.68, 1.69)	0.779
**Behavioral (ref. never or no)**				
Incarcerated, ever	3.62 (1.42, 9.24)	0.007**	3.62 (1.18, 11.13)	0.026*
Smoked tobacco, past 12 months	1.54 (0.97, 2.45)	0.069	0.57 (0.28, 1.13)	0.107
Consumed alcohol, ever	1.43 (1.01, 2.04)	0.047*	0.79 (0.49, 1.28)	0.339
Alcohol dependence^§^	2.84 (1.63, 4.97)	<0.001***	2.61 (1.20, 5.69)	0.016*
Used drugs, ever	3.22 (1.49, 6.96)	0.003**	1.48 (0.58, 3.76)	0.416
Have a doctor	1.61 (0.92, 2.80)	0.095	2.69 (1.32, 5.49)	0.007**
**Comorbidity (ref. never)**				
Diagnosed with diabetes, ever	3.41 (1.75, 6.65)	<0.001***	5.02 (2.35, 10.71)	<0.001***
Diagnosed with HCV, ever	3.30 (0.95, 11.42)	0.059	4.28 (0.85, 21.57)	0.077

Notes. HCV: Hepatitis C virus

* *p*<0.05

** *p*<0.01

*** *p*<0.001

^§^ as measured by the CAGE.

## Discussion

This study provides insight on subpopulations associated with smear positivity. The proportion of smear-positive participants among the incident TB-positive population was low (34.3%). Men, those with diabetes, a history of incarceration, or alcohol dependency have significantly higher odds of smear-positive test results compared to other populations. Those who utilized healthcare services in Almaty city had significantly higher odds of testing smear-positive when compared to those in other study regions.

Previous research has identified several risk factors that are significant for acquiring active TB, including diabetes[25, 26], alcohol abuse[5, 27, 28], and a history of incarceration[3]. This work builds upon those findings by linking these determinants to increased infectiousness, as approximated by sputum smear positivity. Notably, those diagnosed with diabetes were found to have a 5-fold increase in odds of testing smear-positive. It is possible that increased infectiousness may occur as result of the reported correlation between diabetes and impaired host immunity to TB[26]. With 5% of the population currently diagnosed with diabetes, these individuals may significantly contribute to the burden of TB in Kazakhstan[29].

Strong associations between alcohol dependence and incarceration with smear positivity were observed and both have been previously linked to active TB [3, 6, 21, 27, 28]. Exploring TB transmission, due to close contact, confined spaced, and poor ventilation, transmission of TB in prisons is higher than those observed in the general population[3]. Providing routine TB screening and adequate treatment programs within prison systems may reduce overall TB rates and prevent community transmission[3]. TB burden follows a socio-economic status (SES) gradient, with the highest burden among economically underprivileged populations[30, 31]. Participants in our study reported higher rates of unemployment (19.4%) as compared to the national census (5.0%)[32]. This may indicate that our study population experience higher rates of alcohol dependency, history of incarceration, or homelessness compared to the general population, as these factors often coincide with poverty. Together, these factors may partially explain the underlying association between low SES, TB risk, and smear positivity in Kazakhstan. Though TB diagnosis and treatment is free in Kazakhstan, these populations often don’t seek care until their symptoms worsen[33]. This may result in a larger concentration of severe TB cases and increase the observed association between these risk factors and smear positivity in our study.

The utilization of healthcare services in the Almaty area, Kyzylorda, or Kostanay regions appear to be protective of smear positivity as compared to the use of services in Almaty city. The crude deleterious association between region shifts to a protective association once the independent effect of healthcare utilization (for which seeing a physician serves as a proxy) is considered. Urban settings have a higher burden of TB due to higher population density, crowded living and working conditions and lifestyle[31]. Kazakhstan population density is among the lowest in the world, especially in rural settings. Therefore, individuals in Almaty area, Kyzylorda, or Kostanay regions, which are generally rural settings, may have lower odds of smear-positive TB as a result of lower quality diagnostic services and healthcare utilization in rural areas.

Men have an almost 2-fold increase in odds of smear positivity as compared to women. Gendered differences in pulmonary-related health outcomes could be the result of physiological and immune system factors, epigenetics, and differential sociocultural exposure and reporting practices [34–36]. Specific to smear positivity, sputum volume and quality production do not appear to differ by gender [37]. However, gendered reporting differences exist with men receiving more clinical exams for similar coughs [38] and women reporting more coughs but less phlegm (for similar levels of pulmonary distress) [39, 40], a key diagnostic criteria for TB clinical diagnostic algorithms used in Kazakhstan. Similar to regional variations, this potential decrease in phlegm reporting could lead to fewer sputum sample requests and subsequent smear testing and positivity results for women. Additional research into gender quality of clinical service differentials is needed to clarify this relationship and isolate the unique contribution of genetic, physiological and sociocultural factors.

The overall proportion of smear-positive participants among the TB-positive population was low (35%); and slightly lower than previously reported proportions (50%) [41]. This may indicate poor quality sputum sample collection or inadequate test performance by healthcare workers, both of which heavily affects sputum smear microscopy sensitivity[41]. High rates of TB case notification are observed in rural settings, but these populations have significantly lower odds of testing smear-positive. TB case notification rates in Kyzylorda (110.5/100,000) and Kostanay (108.1/100,000) have been reported to be higher than those in Almaty (49.8/100,000)[12]. This may be due to lack of access to accurate and rapid diagnostics tests and reliance of symptom-based diagnosis in rural areas. The utilization of a rapid and sensitive test, like the WHO-recommended Cepheid GeneXpert, may improve the detection and treatment of TB in smear-negative cases while preventing unnecessary risk to patients and conserving resources due to misdiagnosis[42].

There are several limitations to our study. First, TB-positive cases were identified and diagnosed clinically based on symptoms and were unconfirmed by culture, the current diagnostic gold standard for TB. Observed positive associations could be biased towards the null hypothesis, as these TB-negative smear-negative cases would mask true associations. Protective associations, like rural settings, may appear larger than the true effect. Second, unlike previous work[6], this study did not find associations between smear positivity and smoking, drug use, or HIV in our final adjusted model, perhaps owing to our limited sample size (based on geographic and temporal constraints). Our study population also had very low rates of drug use and HIV, which may have prevented us from observing any significant associations. Third, study population was limited to those with access to Kazakhstan’s NTP and who met our inclusion criteria, thus excluding all incident TB-positive patients living alone or without access to TB care. Fourth, the majority of the findings are based on self-report data and open to potential recall and reporting biases. We have found no evidence to suggest that either under or over-reporting of these factors is systematically related to smear positivity status, something many participants may not even be aware of, thus we do not believe that it systematically altered our findings or interpretations.

## Conclusions

The persistence of TB in Kazakhstan suggests the need to expand control efforts to address individual and social determinants of the disease. In our study, men, individuals with diabetes, abuse alcohol, and were incarcerated are at higher odds of testing smear-positive. Active case finding and targeted interventions focused on screening and treating these groups may considerably help reduce the transmission of TB. Integrating TB screening and treatment services with other established health services, like diabetes care, alcohol rehabilitation services, or prisons could greatly improve TB detection rates and treatment adherence and outcomes. Further studies are needed to determine the contribution of smear-negative TB-positive individuals to the spread of the disease in Kazakhstan. Additional studies should be conducted to determine whether interventions specific for populations identified in this study could significantly reduce the spread of TB.
